# Orbital complications of acute rhinosinusitis: a new classification

**DOI:** 10.1016/S1808-8694(15)30130-0

**Published:** 2015-10-19

**Authors:** Antonio Augusto Velasco e Cruz, Ricardo Cassiano Demarco, Fabiana Cardoso Pereira Valera, Antônio Carlos dos Santos, Wilma Therezinha Anselmo-Lima, Regina Maria da Silva Marquezini

**Affiliations:** a Full Professor of Otorhinolaryngology - University of São Paulo - Ribeirão Preto Medical School; b M.S. Attending Otorhinolaryngologist - University of São Paulo - Ribeirão Preto Medical School; c M.S. Attending Otorhinolaryngologist - University of São Paulo - Ribeirão Preto Medical School; d PhD. Professor of Radiology and Image Sciences - University of São Paulo - Ribeirão Preto Medical School; e Associate Professor of Otorhinolaryngology - University of São Paulo - Ribeirão Preto Medical School; f 3rd year otorhinolaryngology resident - University of São Paulo - Ribeirão Preto Medical School. University of São Paulo - Ribeirão Preto Medical School

**Keywords:** orbital abscess, orbital cellulitis, orbital complications, complicated acute rhinusinusitis

## Abstract

Rhinosinusitis is a severe sickness and may have serious complications. Orbital complications happen more often, due to anatomical particularities and are lethal in 5% of patients. They vary from inflammatory signs to proptosis, loss of ocular motility and blindness. **Aim:** We propose a new classification of acute rhinosinusitis complications. **Methods:** A review of 83 patients with CT scan and clinical reports. Patients were evaluated at HCFMRP-USP between 1995 and 2005 and were diagnosed with complicated rhinosinusitis. **Results:** In sixty-six patients, were identified three types of orbital complications: orbital cellulitis (46. 9%), subperiosteal abscess (40. 9%) and orbital abscess (12.1%). Seventeen were considered as eyelid infections and excluded from this new classification system. **Conclusions:** The existing classifications of orbital complications, as Chandler’s, do not consider the orbit’s anatomical characteristics and became obsolete after the development of the CT scan. This study proposes a new, more objective classification to guide the physician in establishing lines of conduct for each case.

## INTRODUCTION

The orbits may be defined as bony compartments, craniofacial pairs, symmetrically located on each side of the nose. Orbit content, made up of the eyes and a whole set of related structures, is intimately associated with the paranasal sinuses, especially the maxillary, ethmoid and frontal sinuses. Anatomically speaking, the orbit is separated from the upper and lower eyelids by the orbitary septum which is the anterior orbit border. This septum is a continuity of the periosteum which lines the orbit walls (also called periorbit), which changes direction at the orbit border, merges with eyelid retraction elements, thus working as a diaphragm, which prevents the anterior bulging of the orbit content^1^.

Acute complications of paranasal sinuses diseases seem to be more frequently in children when compared to adults, and are directly related to the intimate anatomical relations between the paranasal sinuses and other head, neck and chest structures^2^.

Rhinosinusitis is a severe disease that frequently bears severe orbitary complications, which require a fast multidisciplinary approach, because a diagnostic delay may cause the patient’s death. Ognibene et al.^4^ reported an 83.1% of orbit complications in 65 patients studied throughout a 10 year period. Mortimore and Wormald (1997)^5^ found 80% in a population that was studied during 5 years. Before the advent of antibiotic therapy, the prevalence of post rhinosinusitis orbit complications was very high. Some series mention mortality rates between 17-19% and amaurosis prevalence of 20-33%^6^, ^7^. Fortunately, these rates have not gone beyond 5% today.

Both staging and classification of rhinosinusitis are extremely important in order to choose the correct therapeutic approach. Currently a terminology analysis regarding the classification of orbital infections of sinusal origin is confusing and inaccurate. If the term cellulitis, broadly employed to call any type of orbital infectious involvement is technically correct, the same cannot be said about the pre-septal adjective that by definition expresses something, which does not go beyond the orbit. Thus, the expression “pre-septal cellulitis” used by some authors^5^, ^6^, ^7^, can only make sense to discuss eyelid infection and never orbitary infection. There are also similar problems regarding the terms “periorbitary” and “retro-orbitary” cellulitis, commonly used in clinical practice to name intraorbitary infections. In a similar fashion, “cavernous sinus thrombophlebitis” cannot be a category of orbitary cellulitis, for the simple reason that the cavernous sinus is an intracranial structure, and not orbitary.

The goal of the present investigation was to review CT scans of patients with acute rhinosinusitis complicated by orbitary infection and, based on CT findings, propose a more coherent classification for the different modes of orbitary cellulitis.

## MATERIALS AND METHODS

The present study was approved by the Ethics in Research Committee, according to protocol # 1930/97.

We reviewed the CT scans and charts of 83 patients, with ages varying between 26 days and 77 years of age, seen at the University Hospital of the Ribeirão Preto Medical School - USP, between 1995 and 2005. In all the CT scans, we ordered coronal and axial views of the orbit and paranasal sinuses, with 3mm slices before and after injecting endovenous contrast. An ophthalmologist, a radiologist and a rhinologist carefully assessed the images from each patient, and there was absolute agreement among them.

## RESULTS

We detected three main types of intra-orbitary alterations in 66 patients:

a) diffuse infiltration;
b) subperiosteal abscess;
c) orbitary abscess. Seventeen cases were treated as eyelid infections.

**Figure 1:**
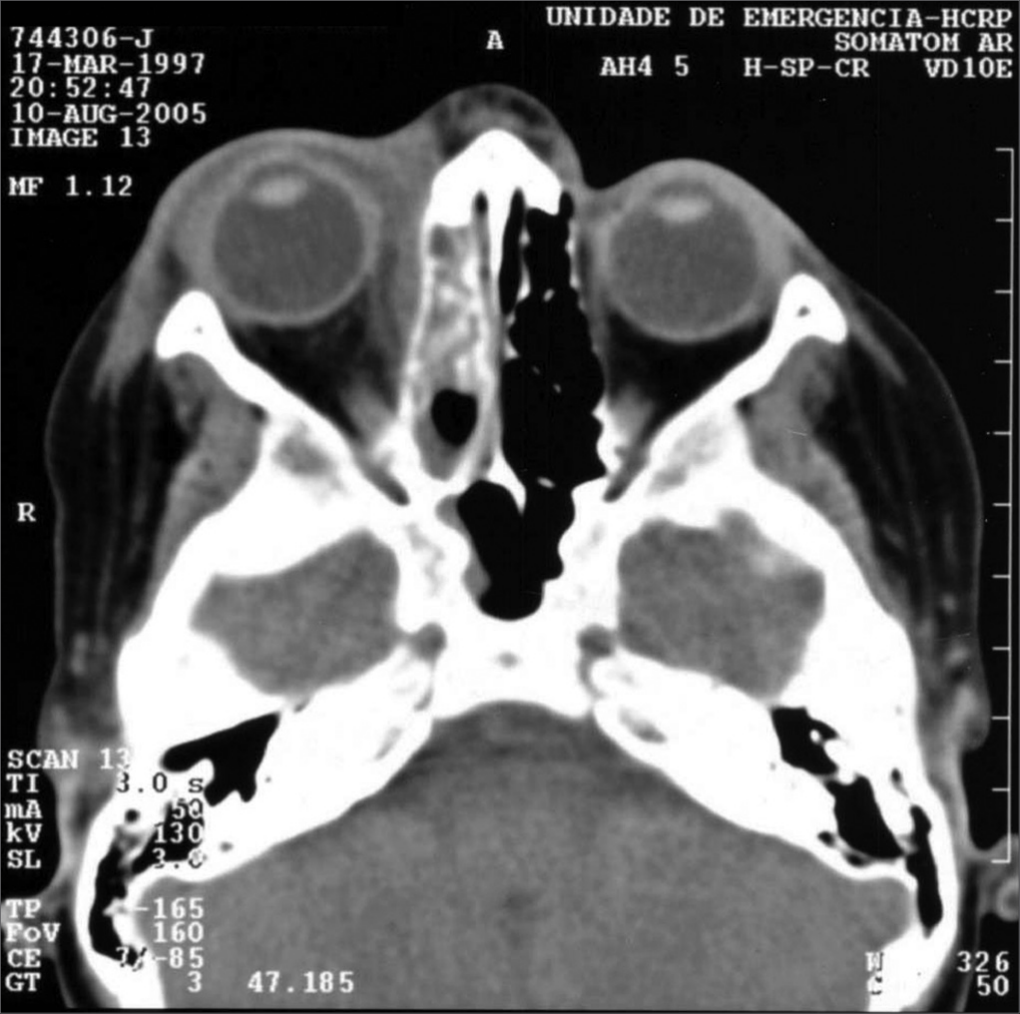
Axial view CT scan of the orbit showing orbitary cellulitis on the right side.

Diffuse fat infiltration was characterized by an increase in extra or intraconal fat density. Transition limits between normal fat and high-density fat were typically gradual and not well defined (Figure 1). This diffuse cellulitis was seen as an isolate characteristic in 31 patients (46.9%), with ages varying between 2 months and 71 years, affecting the right orbit in fifteen cases (48.3%). Nine patients did not have a favorable outcome in 48 hours with endovenous antibiotic, thus a surgical drainage was carried out.

**Figure 2:**
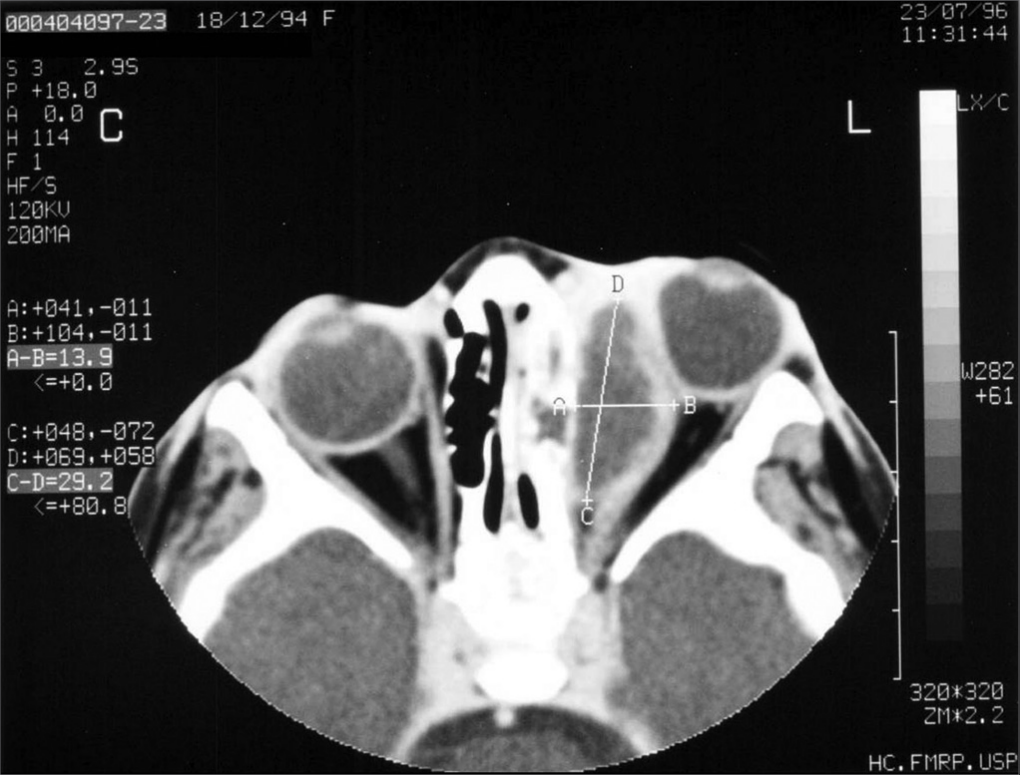
Axial view CT scan of the orbit showing subperiosteal abscess on the left side.

Subperiosteal abscess was diagnosed when the periorbit was elevated, in at least one orbitary bony wall adjacent to the paranasal sinus (twenty-seven cases - 40.9%, with ages between 1 and 77 years; 48.1% of them on the right side orbit) (Figure 2). The detached periosteum was well outlined, defining the border of the fluid collection. Initially, the patients were admitted to the hospital and treated with endovenous antibiotic. An ophthalmologist thoroughly assessed the patients in relation to signs of inflammation, eye movement, proptosis, and mainly visual function. Patients with visual deterioration or no improvement in their clinical picture within 12 to 24 hours, had their abscesses drained (66.6% of the cases).

**Figure 3:**
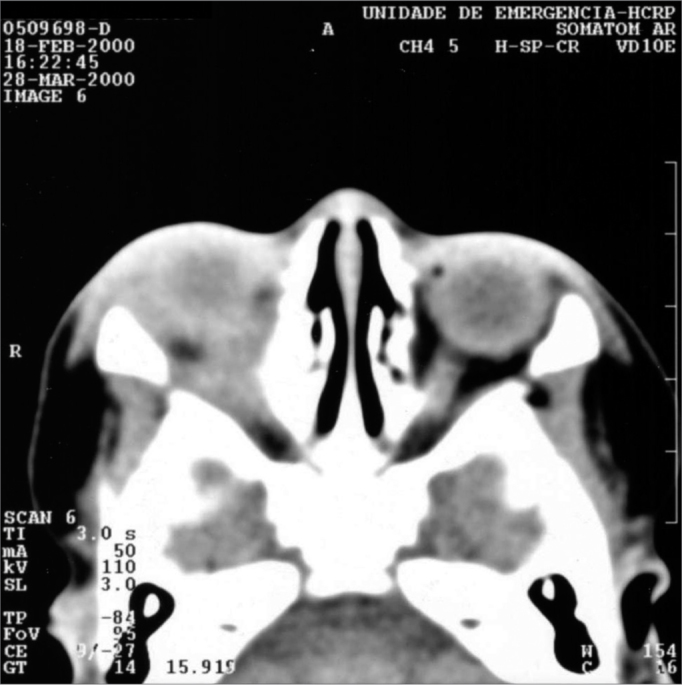
Axial view CT scan of the orbit showing orbitary abscess on the right side.

**Figure 4:**
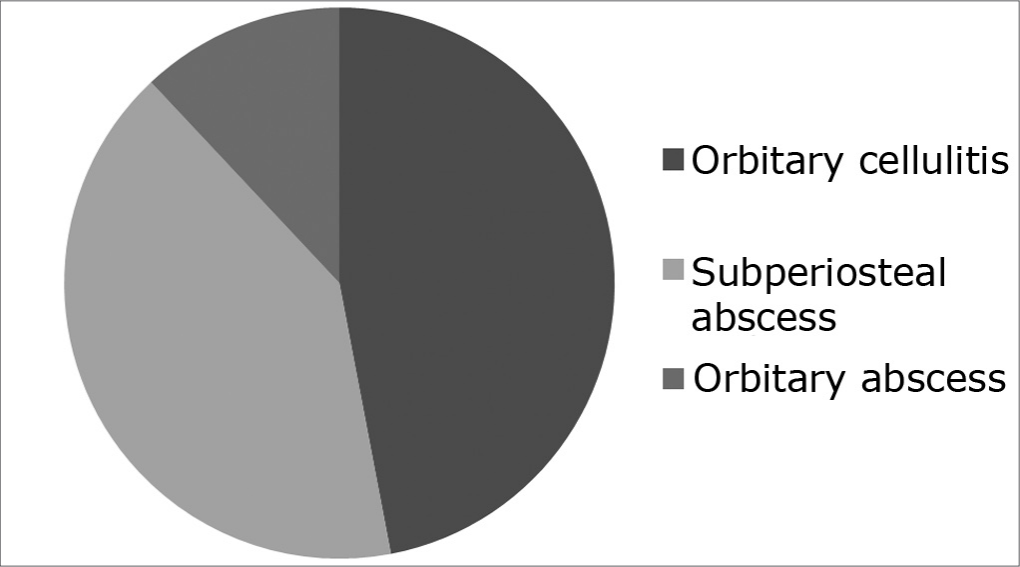
Frequence of orbitary complications.

Whenever we found heterogenous density (with or without a circle aspect) within orbit fat, we called it an orbitary abscess. (Figure 3). Eight cases had it(12.2%), with ages varying between 26 days and 77 years, more frequently on the right orbit (50%). These patients underwent surgical drainage of their abscesses (Figure 4).

Thus, we propose a new classification in accordance with Table 1. Of the sixty-six cases, forty-three (65.1%) were males. Both right and left orbits were equally involved, and two were bilaterally affected.

**Table 1:** Orbitary complications of acute rhinosinusitis.

I - ORBITARY CELULITIS
II - SUBPERIOSTEAL ABSCESS
III - ORBITARY ABSCESS

## DISCUSSION

Since 1937, when Hubert^8^ published the first classification of rhinosinusitis complications, until 1977, when Mortimore and Wormald^5^ proposed a new classification for orbitary complications, a major confusion set in the nomenclature of orbitary infections, shown by the fact that the authors used the terms pre-septal or periorbitary, which by definition are related to extra-orbitary pathologies, and are used to stage intraorbitary infections.

In 1937, Hubert^8^ published a classification that included eyelid, orbital and intracranial diseases. According to the author, they could be staged as: I- eyelid inflammatory edema, II- orbit subperiosteal abscess, III- diffuse orbitary celulitis, IV- orbitary abscess and V- cavernous sinus thrombophlebitis. The latter is obviously not a form of orbitary cellulitis, because this is an intracranial complication of sinusitis and should not be classified as a subtype of orbitary cellulitis. Hubert’s description of group I was confusing, including orbitary and eyelid signs in the same category. Textually, se says: “in the first group the infection is confirmed in the sinus and there is only an inflammatory edema on the eyelid… the edema must; therefore, extend to the orbitary tissue. When this happens, there is exophthalmia and eyeball movement limitation”. In 1948, Smith & Spencer^9^ kept the same impression of group I, however they defined the category as eyelid inflammatory edema with or without orbit edema. In 1970, Chandler et al.6 used the classification from Hubert, Smith & Spencer. It must be noted that Chandler kept the Group I definition, however perceiving the theoretical inconsistence of labeling an eyelid edema as a category that includes exophthalmia and eye mobility restriction, he removed the word eyelid from this category. Chandler also modified category III definition. While Smith thought it was due to orbitary vein phlebitis, Chandler used the expression “diffuse cellulitis” in order to describe inflammatory cells infiltrating orbitary fat tissue.

Chandler et al’s original classification.^6^, published in 1970, has been the one most employed since then, and divides orbitary celulitis in the following categories:

1) inflammatory edema,
2) orbitary cellulitis,
3) subperiosteal abscess,
4) orbit abscess and
5) cavernous sinus thrombosis.

The term pre-septal used to name Chandler’s category I first appeared in the literature after the work from Moloney et al.^10^. This expression is used to describe eyelid pathologies and must be abandoned for orbitary infections description. The last one of Chandler’s characteristics (cavernous sinus thrombosis) is also not related to any orbitary structure; however, it mentions one of the most feared intracranial complications of cellulitis, and, therefore, should not be considered as a type of orbitary cellulitis.

The so-called “Groote Schuur Hospital” classification published by Mortimore and Wormald^11^ increases the confusion of terms in the staging and classification of the different forms of orbitary celulitis. The authors insist in using the expression “pre-septal” to call something that, by definition, is retroseptal. The authors divide the genuine orbitary infections (post-septal) in subperiosteal and intraconal. This division is not real. If the subperiosteal category is true, the same cannot be said about the use of the term intraconal as a synonym of post-septal. Technically, the term “intraconal” means: within the space outlined by the extra-ocular muscles. Behind the septum, there is more than just this space, there is the extraconal space, which is defined by the compartment existing between the cone and the periorbit. The classification still bears a severe error as it considers as interchangeable the following expressions: “localized post-septal cellulitis” and “orbitary apex syndrome”. The meaning of the latter is only to localize, that is, it encompasses any process that may be happening in the upper orbitary fissure, and not necessarily infection. On the contrary, the main etiologies of the orbit apex syndrome are of autoimmune origin, tumoral or traumatic.

New diagnostic means, such as CT scan, MRI and nasal endoscopy contribute to the early diagnosis and help identify the complication stage, making it possible to instate a more efficacious therapy. Therefore, after a detailed history and physical exam, image technology is fundamental^12^.

CT provides a tridimensional assessment of the abscess size, as well as a clear relation with the eyeball, extrinsic muscles and optic nerve. In children, it is critical to distinguish between orbitary cellulitis and subperiosteal abscess. According to Clary et al.^13^, the correlation between radiologic and surgical findings, although not absolute, points to CT scan as the paramount means of diagnosis. In cases of subperiosteal abscesses, CT scan reveals an edema of the extrinsic eye muscles and a homogeneous opacification between the orbit walls and the shifted periorbit, adding to it all, the eyeball is shifted in a non-axial fashion. In case of orbitary abscesses, CT reveals obliteration of the extrinsic muscles and the optic nerve; and a homogeneous mass matching the signs of an abscess.

## CONCLUSION

CT scan may detect all diffuse infiltration of periorbitary fat, periorbit shifting or a true orbit abscess in all cases. We propose a new and simple classification, based on more specific terms to help the physician establish the approach to each case in a linear fashion. The expression “pre-septal” referring to orbitary cellulitis is inaccurate and must be abandoned.
